# Th17 Cells and Cytokines in Leprosy: Understanding the Immune Response and Polarization

**DOI:** 10.1590/0037-8682-0265-2023

**Published:** 2023-10-30

**Authors:** Larissa Marchi Zaniolo, Amílcar Sabino Damazo

**Affiliations:** 1 Universidade do Estado de Mato Grosso, Faculdade de Ciências Agrárias, Biológicas, Engenharias e da Saúde, Tangará da Serra, MT, Brasil.; 2 Universidade Federal de Mato Grosso, Faculdade de Medicina, Programa de Pós-Graduação em Ciências da Saúde, Cuiabá, MT, Brasil.; 3 Universidade Federal de Mato Grosso, Faculdade de Medicina, Departamento de Ciências Básicas em Saúde, Cuiabá, MT, Brasil.

**Keywords:** Immune response, Interleukin-17, Leprosy, Leprosy reaction

## Abstract

While there are conflicting data concerning interleukin (IL)-17 levels in the serum of patients with leprosy compared with those in healthy controls, higher levels have been more evident in the tuberculoid clinical form of leprosy and type 1 reactions. This review aimed to highlight the role of Th17 cells and their cytokines in leprosy. Cytokines such as IL-1β and IL-23 induce Th17, while transforming growth factor beta and IL-10 inhibit Th17, indicating that the balance between Th17 and regulatory T cells is crucial for leprosy polarization. However, more comprehensive paired studies are required to better elucidate the role of Th17 cells in leprosy.

## INTRODUCTION

Leprosy is a transmissible, chronic disease that affects the skin and nerves. It is caused by *Mycobacterium leprae (M. leprae)* and *Mycobacterium lepromatosis (M. lepromatosis)* infection, and can lead to physical disability and deformities^1^. Individual susceptibility depends on environmental, genetic, and immunological factors^2^.

The clinical forms of leprosy can be classified into two categories: tuberculoid-tuberculoid (TT), with a predominantly cellular immune response, and lepromatous leprosy (LL), with a predominant humoral response. Other clinical forms of leprosy, such as borderline tuberculoid (BT) leprosy, borderline-borderline (BB) leprosy, and borderline leprosy (BL), show more unstable immunological characteristics ranging across both polar forms^3^ ^-^ ^7^. Additionally, patients may experience acute immunological reactions before, during, or after pharmacological treatment, known as type 1 reactions (RT1), resulting from a sudden increase in cellular immunity, or type 2 reactions (RT2), which usually occur in clinical form with a high bacillary load owing to the deposition of immune complexes with Bacillus antigens^8^.

The immune response varies depending on the clinical form of the disease. Conditions that exhibit an intense cellular response with type 1 helper T cells (Th1) and cytokines such as interleukin (IL)-2, IL-12, tumor necrosis factor-alpha (TNF-α) and interferon gamma (IFN-γ) can be identified that culminate in bacillary load control. For example, this can be observed in some patients with the clinical form of TT. However, other patients may exhibit an intense humoral response with Th2 cells and cytokines IL-4, IL-5, and IL-10 and transforming growth factor beta (TGF-β), leading to inhibition of macrophage microbicidal activity and bacillary proliferation. Patients with the LL form present with this latter response. 

Other T lymphocyte populations have been implicated in the immunopathology of leprosy, such as Th17. Similar to Th1 cells, Th17 cells produce proinflammatory cytokines, mainly IL-17, IL-21, and IL-23. Higher levels of IL-17 have been observed in patients with the TT clinical form, contributing to maintenance of the cellular immune response, increasing the number of Th1 effector cells, recruiting neutrophils^9^ ^-^ ^14^, and promoting tissue inflammation in skin lesions^11^ ^,^ ^15^. In addition, some studies have reported the involvement of IL-17 in the pathogenesis of RT1^12^ ^,^ ^16^ ^-^ ^18^ and RT2^19^.

Th17 cells have IL-6 and TGF-β as differentiation factors, along with transcription factors STAT3, RORγt, and RORα^20^, and produce inducible nitric oxide synthase (iNOS), which acts in *M. leprae* destruction in phagosomes^21^.

Moreover, the upregulation of cytokines associated with Th17 cells leads to suppression of T regulatory cells (Tregs), which play a crucial role in maintaining peripheral tolerance. A heightened abundance of Tregs has been linked to immunosuppression and enhanced proliferation of bacilli in patients with LB and LL^12^. The equilibrium between Th17 and Treg populations has previously been described. A decline in Tregs is concomitant with heightened Th17 activity, decreased TGF-β levels, and elevated IL-6 and IL-21 levels^15^. Additionally, IL-10 and TGF-β inhibition in patients with LL leads to the restoration of Th17 cell presence, underscoring the potential manipulation of the cytokine milieu to benefit the patient's condition.

As leprosy presents as a complex immunoregulatory scenario, this review aimed to advance knowledge concerning Th17 cells and their cytokines, and highlight their participation in the immune response to leprosy.

### Research strategy and selection criteria

**FIGURE 1:**
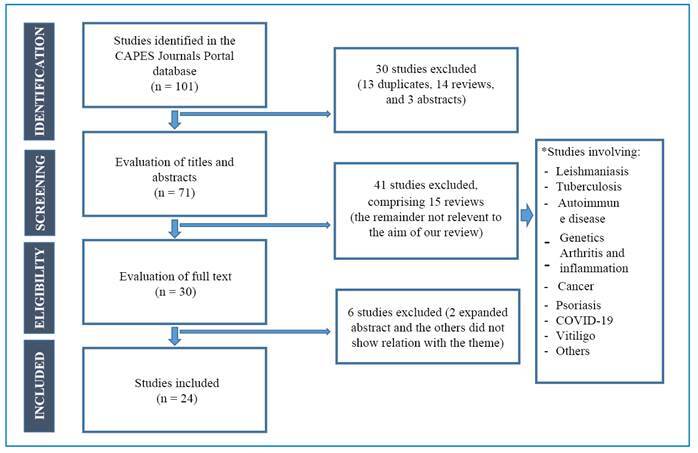
Flowchart of included studies.

Relevant studies were retrieved from the CAPES Journal Portal in March 2023 using the following descriptors: “leprosy” and “Th17,” with the Boolean operator “AND.” Full articles addressing the pathophysiology of leprosy related to the Th17 immune response profile in the clinical form and reactional state were included ( Supplementary Table 1). Studies involving leprosy and other autoimmune and immunosuppressive diseases, as well as reports, abstracts, reviews, and duplicate records were excluded (Figure 1).

### Th17 levels in the clinical forms of leprosy

In BL, Th17 cells play a protective role in the immune response^13^ ^,^ ^22^ secreting IFN-γ, IL-4 and IL-5^13^. Conflicting data concerning IL-17 levels have been reported, suggesting decreased levels in the serum of patients with leprosy^23^ ^,^ ^24^, whereas other studies^13^ ^,^ ^14^ ^,^ ^17^ ^,^ ^25^ have reported higher levels in the TT/BT polar forms than in the LL form^11^ ^-^ ^14^ ^,^ ^25^ ^,^ ^26^. Furthermore, one study concerning IL-17F identified higher levels in non-reactional patients than in healthy controls, suggesting the greater the bacillary load, the greater the secretion of IL-17F^17^.

One study compared the differences between peripheral blood mononuclear cells (PBMCs) and the immune response at the site of skin injury and reported generally higher levels of IL-17, IL-21, IL-22, IL-23, MMP13, CCL20, and CCL22 and lower levels of IL-1β and RORC transcription factor in patients with TT compared with patients with LL^13^. Notably, no difference in skin lesions was observed in relation to differences between IL-23 and IL-6 levels^13^. Azevedo et al.^26^ obtained similar data regarding IL-17A and IL-21 levels, but differences were observed in IL-22 and IL-23 levels. Furthermore, Wang et al.^27^ reported that the IL-17 levels in leprosy skin lesions were similar to those in healthy controls. Another report showed no difference in serum IL-22 levels among the clinical forms of leprosy^16^. These study findings highlight the challenges in determining Th17 predominance in these patients.

### Involvement of IL-23 in Th17 cell differentiation

The cytokine IL-23 is secreted by keratinocytes, epithelial cells, and macrophages, and has been found in higher levels in patients with TT compared with those with LL, but at lower levels than those in healthy controls^13^ ^,^ ^22^. Blocking the signaling of rIL-23 *in vitro* in CD4+ T cells has been reported to decrease IL-17A production, increase TGF-β levels, and have minimal effect on IFN-γ and FoxP3 levels^22^.

Cells stimulated with phytohemagglutinin (PHA) and BCG taken from patients with paucibacillary (PB) leprosy had higher levels of TCD4+ IL-17+ and TCD4+ IFN-γ+ compared with patients with multibacillary (MB) leprosy^10^ . The inflammatory cytokines IFN-γ and IL-17 exhibited a positive correlation in these patients, suggesting that they act synergistically in controlling the infection. Unlike in patients with PB-type leprosy, those with MB-type leprosy showed changes in their immune response profile after multidrug therapy (MDT), showing an increase in TCD4+ IL-17+^10^.

Concerning IL-17A gene polymorphism, genetic factors influence the type of host immune response against the bacillus. Farag et al.^25^ showed that, while IL-17A gene polymorphisms were not significantly associated with genetic susceptibility to leprosy, the GG genotype and G allele of IL-17A were associated with progression to the LL form.

IL-26 is another cytokine secreted by Th17 cells that contributes to defense against bacilli within the cells. When added to macrophages *in vitro*, IL-26 enters cells infected with *M. leprae*, decreasing their viability, inducing autophagy, and promoting the fusion of phagosomes containing bacilli with lysosomal compartments. IL-26 is more highly expressed in BT lesions, granulomas, and near lymphoid cells, and is diffusely expressed in mononuclear myeloid cells^28^.

### Th17 in leprosy reactions

When comparing two reactive states, Yuniati et al.^29^ identified higher levels of CD4+ RORg-Th17 and IL17 in an RT1 group compared with an RT2 group. Another study showed that IL-17 levels were higher in patients in an RT1 group and that Th17 cells were positive for both IL-17A and IL-17F^18^. Chaitanya et al.^17^ identified higher IL-17F levels in the blood of patients with RT1 compared with levels in non-reactive and healthy controls and in patients with RT2s compared with controls, suggesting that IL-17 plays a role in the regulation of leprosy reactions.

Vilani-Moreno et al.^30^ evaluated RT2 leprosy at the beginning of the reactional state and one month after treatment. No differences in IL-17, IL10, IL-4, IL-2 or IFN-γ levels were observed. However, higher levels of nitric oxide, TNF-α, and IL-6 were observed at the onset of the reaction. IL-6 levels decreased after treatment, suggesting that IL-6 may be a potential biomarker. Another study compared patients with RT2 and patients with LL and reported reduced levels of TCD4+ cells, FoxP3, and IL-10 levels in the RT2 group, but higher levels of IL-4, TNF-α, IFN-γ, IL-1β, IL-6, IL-8, and IL-17A^31^ ^,^ ^32^.

Other cytokines may also be involved in the cell differentiation pathways of Th17 cells in the reactive state. Higher IL-17A/F, IL-21, IL-22, and IL-23 levels, and higher chemokine CCL20 and CCL22 levels have been reported in reactive cells compared with their non-reactive counterparts^15^. Supporting these data, another study identified high IL-21 levels in both peripheral blood and skin lesions in patients with RT1 compared with non-reactive patients. Furthermore, IL-21 gene expression in multilocus sequence analysis-stimulated PBMCs showed a significant correlation with IL-17A/F and RORC, but not with IFN-γ, TGF-β, and FoxP3, suggesting that elevated IL-21 participates in the pathogenesis of RT1 through inducing differentiation and increased function of Th17 cells^33^. Higher serum IL-22 levels have been reported in patients with TR2 compared with healthy controls^16^.

Saini et al.^34^ reported elevated IL-17A, IL-23, IL-6, and rIL-6 levels in patients with RT1. Through blocking rIL-6 and rIL-23, IL-17A expression decreased and TGF-β expression increased, confirming the roles of these cytokines in leprosy. Castro et al.^35^ suggested that the upregulation of IL-17 and IL-6 influences the development of RT2. Additionally, Azevedo et al.^26^ reported higher expression of IL-17F, IL-8, CCL20, and CCL29 in patients with RT2 and lower expression of IL-22, IL-23, TGF-β, and FoxP3.

Studies involving reactive patients have identified an increase in TGF-β, INF-γ, and IL-17 levels in patients with RT2 compared with those with RT1 and non-reactive patients^36^. These data differ from those reported by Gomes de Castro et al.­^35^ who found lower levels of these cytokines in patients with RT2 than in those with RT1 and LL, suggesting their role in maintaining a hyporesponsive state in the LL polar type, which may lead to the genesis of leprosy reactions. However, these divergent data emphasize the importance of further investigations with regard to TGF-β along with other cytokines responsible for the differentiation of Th17 cells, such as IL-6, IL-21, and IL-1β.

Santos et al.^14^ reported that patients with MB-type leprosy had lower IL-17A and IL-1β levels than those with PB-type leprosy; however, those who evolved to reactional states had higher serum concentrations of IFN-γ, indicating an association of Th1 cells both in patients with the PB type and with leprosy reactions. In contrast, Th17 cells have been associated with a more effective inflammatory response in patients with PB-type leprosy but not as predictors of reactions in patients with MB-type leprosy.

In a cohort of patients with RT2 who were treated with thalidomide for 21 days, a reduction in IL-20 and an increase in IL-21 and FoxP3 were observed, while IL-17 was observed before and after treatment, confirming that these cells are involved in RT2 and are regulated by thalidomide, suppressing inflammatory components and enhancing cellular immunity mediated by this profile^37^.

### The relationship between Th17 and Tregs in the immune response to leprosy

Several studies have investigated the cytokines involved in Treg and Th17 cells in leprosy, indicating that the balance between these cells appears to be involved in the development of vigorous responses to *M. leprae* ^11^ ^,^ ^12^ ^,^ ^14^ ^,^ ^38^. Decreased Treg suppression is associated with increased Th17 activity. However, elevated Th17 profile cytokines contribute to inflammation in the affected area, resulting in tissue and nerve injury^15^ ^,^ ^26^ ^,^ ^29^ ^,^ ^36^. Furthermore, Th17/Tregs were mainly distributed within and around the granulomas in BT/TT. In contrast, they were observed around macrophages in BL/LL, suggesting an interaction with histiocytes^27^.

TGF-β participates in the differentiation of Treg FoxP3 and inhibits Th17, as reported by Saini et al.^15^ In cutaneous lesions, using immunohistochemistry, Wang et al.^27^ found that FoxP3 expression was higher than that in healthy controls. Quaresma et al.^11^ identified a higher TGF-β expression in patients with LL and Sadhu et al.^12^ reported higher IL-10 levels in patients with BL/LL compared with those with the TT form. De Castro et al.^35^ also showed higher IL-10 levels in BL than in LL. These findings are in accordance with those reported by Attia et al., who showed higher levels of TGF-β in patients compared with controls, although no differences were observed across the leprosy spectrum^16^. Increased IL-10 production is linked to reduced IL-17 production, which contributes to the progression of MB-type leprosy.

In addition, Sadhu et al.^12^, in their study in India with patients using flow cytometry on peripheral blood stimulated with *M. leprae*, observed a five-fold higher frequency of Tregs producing IL-10 in the BL/LL types, whereas Th17 cells had higher levels in the BT/TT types, showing an inverse correlation between the presence of IL-10 and IL-17 in these polar forms. Blocking IL-10 and TGF-β resulted in the re-establishment of IL-17+ T cells in BL/LL. Moreover, the action of inducing cytokines TGF-β, IL-6, and IL-23, or the secretory cytokine IL-17 by Th17 cells decreased the numbers of FoxP3 + Tregs and increased those of CD4+ IL17+^12^. One study showed that the plasticity of FoxP3 + Tregs can be manipulated according to the environment, using cultured cells stimulated with *M. leprae* and/or IL-12, IL-23, rIL-2, and anti-CD3/CD28, where rIL-12 and rIL-23 significantly reduced the production of TGF-β and IL-10 by Tregs, and IL-12 converted FoxP3+ Tregs into Th1-like cells, increasing pStat4 levels in Tregs and IFN-γ production, while IL-23 converted Tregs into Th17-like cells, increasing pStat3+ and IL-17A+^38^.

These findings indicate that the balance between Treg and Th17 cells is important for host immunity, and that manipulation of the cytokine environment can promote cellular responses, potentially being useful as a therapeutic approach for patients with the LL polar form to achieve parasite control without inducing immunopathology^12^ ^,^ ^38^.

**FIGURE 2:**
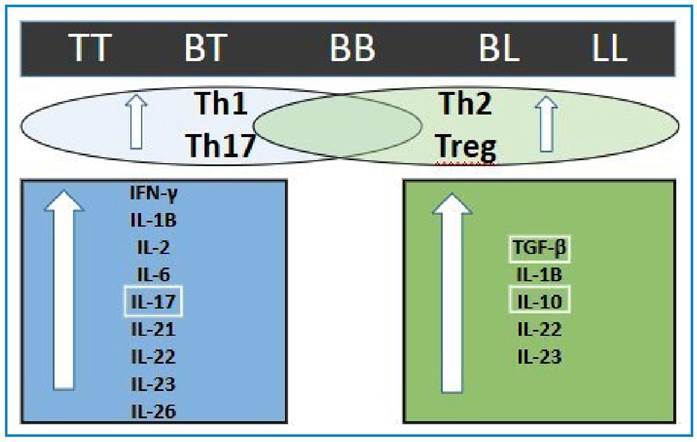
Immunological characteristics of the Th17 profile presented in the clinical forms of leprosy.

Abbreviations: BB: borderline-borderline; BL: borderline leprosy; BT: borderline-tuberculoid; IFN-γ: interferon gamma; IL: interleukin; LL: lepromatous leprosy; TGF-β: transforming growth factor beta; Th1: type 1 helper T cells; Th2: type 2 helper T cells; Th17: type 17 helper T cells; Treg: T regulatory (cells); TT: tuberculoid-tuberculoid.

Figure 2 summarizes the importance of these immune cells and their cytokines in leprosy. 

## CONCLUSION

The role of Th17 cells in the immune response to *M. leprae* and *M. lepromatosis* infection in leprosy requires further elucidation. Higher IL-17 levels appear to occur in leprosy, predominantly in relation to the TT polar form and TR1. Additionally, obtaining a balance between opposing Th17 and Tregs is essential for both leprosy polarization and the development of reactional states.

However, more comprehensive paired studies using skin lesions and blood samples that compare between healthy controls and patients with and without reactions are needed to better elucidate the effects of Th17 cells and their secretion and inducing of cytokines.
