# Supplementary material for: Th17 Cells and Cytokines in Leprosy: Understanding the Immune Response and Polarization
Source: Rev Soc Bras Med Trop. 2023 Oct 30;56:e0265-2023. doi: 10.1590/0037-8682-0265-2023 (PMC10615336; doi:10.1590/0037-8682-0265-2023)
Supplement: Supplementary file 1 [file 1678-9849-rsbmt-56-e0265-2023-supp1.pdf]

Table 1 supplementary. General data of the articles included highlighting the type of sample used, cell types and cytokines.

Author/Year;	N; clinical form	Type of sample / test / cytokines / cells
SHI et al., 2022	N=27/28<br><br>HD=5, TT=11 and LL=12.	SKIN;<br>Blocking with anti-IL-23R, ELISA (keratinocytes / IL-17A); flow cytometry (CD3, CD4, IL-23R, IL-17A, IFN-γ, TGF-β, FoxP3), PCR (IL-23R, IL-17A, IL-6R).
SAINI et al., 2016	N= 66/68<br><br>BT=19; LL=19, RT1=15 and RT2=15.	BLOOD;<br>Cells with and without stimulation by MLSA, FLOW CYTOMETRY (IL-17A, CD3, CD4, CD25, CCR6, FOXP3, TGF-β and STAT3), PCR (cell surface marker - CD28, CD34, CD3D, CD3E, CD3G, CD4, CD40LG, CD8, ICAM1, ICOS, ISG20, cytokines- CSF2, CSF3, IFN-γ, IL-10, IL-12B, IL-13, IL-15, IL-17A, IL17C, IL-17D, IL- 17F, IL-18, IL-1β, IL-2, IL-21, IL-22, IL-23A, IL-25, IL-27, IL-3, IL-4, IL-5, IL-6, TGF-β1, TNF, cytokine receptor-IL12RB1, IL12RB2, IL17RB, IL17RD, IL17RE, IL23R, IL7R, CHIMOCINES-CCL1, CCL2, CCL20, CCL7, CD247, CX3CL1, CXC L1, CXCL12, CXCL2, CXCL5, CXCL6, IL8, MMP13, MMP3, MMP9 transcription and signaling markers - CACYBP, CEBPB, CLEC7A, S1PR1, FOXP3, GATA3, JAK1, JAK2, NFATC2, NFKB1, RORC, SOCS1, SOCS3, STAT3, STAT4, STAT5A, STAT6 , SYK, TBX21, TIRAP, TLR4, TRAF6, YY1) and ELISA (IL-17A/F, IL-21, IL-22, IL-23A, IL-6, IL-1β, IFN-γ and TGF-β) .
SAINI et al., 2020	N=58<br><br>BT=29 and RT1=29<br><br>FLOW CYTOMETRY BT=10/RT1=10	BLOOD;<br>Cell culture with and without MLSA stimulation; Blocking with IL-6R and IL23R, FLOW CYTOMETRY (CD3, CD4, IL-6R, IL-23R, IL-17A, FOXP3, TGF-β and IFN-γ); PCR (IL-6R, IL23R, IL-1β, Stat3, TNF-α, CCL22, RORC, JAK1, IL-2, IL-17F, JAK2, CCL20, IL-17D, IL-23, IL-17A, IL-23R, IL-10, IL-17C, IL-6, IL-25, IL-21, IL-22); ELISA (IL-17A).
ATTIA et al., 2014	N=83<br><br>HCT=40<br>TT=6, PNL=5, borderline=9 (BT=5, BB=3, BL=1), LL=11, RT1=6 and RT2=6.	BLOOD;<br>FLOW CYTOMETRY (CD25, CD4 and FoxP3) and ELISA (IL-10,17 and IL-22, TGF-β and Tregs).
MARTINIUK et al., 2012	RT2=7	SKIN;<br>PCR (Primers - hRORγT (16); hCD70 ; hCD27; hIL-17A (IL-17); hIL-17B (IL-20); hIL-17C (IL-21); hIL-17D (IL-27); hIL -17E (IL-25); hIL-17F (IL-24); hPLZF-1; hCTLA4, hAHR, hIL22, hiNOS2, hARNT, hIDO, hGARP, hIL9, hCD46 and hFoxP3).
COSTA et al., 2018	N=114<br><br>NR=40<br><br>RT1=56 (TT=3; BT=28; BB=2; BL=22, LL=1),<br><br>RT2=18 (BL=9, LL=9).	SKIN;<br>IMMUNOHISTOCHEMISTRY (cells= CD25/Foxp3, cytokines= Treg, TGF-β, IFN-γ, IL-17), HE and FITE-FARACO.<br><br>(Treg - RT1=18, RT2=12 reactional=20 and NR=20)<br>(TGF-β, IL-17, IL-10 and IFN-γ – TR1=12, TR2=12)
DE ALMEIDA-NETO et al., 2015	N=33<br><br>PB=10, MB=13 (I=4, TT=6, BB=7, BL=2, LL=4) and HHC=10/7.	BLOOD;<br>Cells stimulated with PHA and BCG; IMMUNOHISTOCHEMISTRY (TH17 cells (CD4+ IFN-IL-17+).
SADHU et al., 2016	N=50<br><br>BL/LL=20, BT/TT=20 and HHC=10.<br><br>For identification of Treg n=40 (BT/TT=15, BL/LL=15 and HC=10)	BLOOD;<br>WCL stimulated cells; IL-10/TGF-β blockade (LL=5), FLOW CYTOMETRY.<br>Cytokines: IL-10, IL-17, IFN-γ and FoxP3, cell surface markers CD4, CD25, CD45RO, PD-1, PDL-1, CCR4, CCR5 and CCR6, Recombinant soluble proteins of TGF-β, IL-6, IL-17, IL-22 and IL-23.
SANTOS et al., 2017	N=74	SKIN/BLOOD;<br>HE, IMMUNO-FLUORESCENCE (skin= IFN-c, IL-17 or IL-10), FLOW

	HHC=23<br><br>MB=28, PB=23<br>(I=9, TT=14, BT/BB/BL=12 and LL=6)<br><br>RT1=6 and RT2=2	CYTOMETRY (IL-12p70, IFN-γ, IL-17A, IL-1β and IL-10).<br>Cell surface markers - CD3, CD4 and CD8 and intracellular cytokines - IL-17A and IFN-γ (TT=5, LL=6 and HC=6).
SAINI; RAMESH; NATH, 2013	N=46<br><br>HHC=5<br>HCT=4 (plastic surgery)<br><br>BT=19 and LL=18	SKIN/BLOOD;<br>Cells stimulated with PHA and MLSA, FLOW CYTOMETRY (CD3, CD4, CD8, IL-17A/F, IL-21, STAT3), ELISA (blood/ IL-17A/F, IL-21, IL-22, IL-23A, IL-6, IL-1b, IFN-γ and IL-5) and PCR (skin / IFN-γ, IL-4, IL-5, IL-6, IL-6R, IL-2, IL-27 , IL-17A, IL-17C, IL-17D, IL-17F, IL-1β, IL-21, IL-22, IL-23A, IL-23R; chemokines: MMP13, MMP3, CCL20, CCL22; signaling molecules and transcription factors: RORC, SOCS1, STAT3).
WANG et al., 2018	N=75<br><br>BL=15, BT=14, LL=16, TT=15 and HC (plastic surgery)=15	SKIN;<br>IMMUNOHISTOCHEMISTRY (RORγt, IL-17, FOXP3, IL-10 and TGF-β).
YUNIATI et al., 2017	N=50<br><br>RT1=27 and RT2=23	SKIN;<br>HE, IMMUNOHISTOCHEMISTRY<br>(CD4, C25, Th17, FOXP3, RORgT, TGF-β1).
AZEVEDO et al., 2017	N=97<br><br>TT=14, BT=15, BB=14, BL=12 and LL=10.<br><br>RT1=10 and RT2=12.<br><br>HC (plastic surgery)=10	SKIN/BLOOD;<br>PCR (17 chemokines, CCL1, CCL2, CCL3, CCL5, CCL11, CCL17, CCL18, CCL20, CCL22, CXCL1, CXCL2, CXCL9, CXCL10, CXCL11, CXCL12, CXCL16, CX3CL1; the cytokines (24): IL-1A, IL -1B, IL-1RN, IL-2, IL-4, IL-5, IL-6, IL-8, IL-9, IL-10, IL-12A, IL-12B, IL-13, IL-15 , IL-17A, IL-17F, IL-18, IL-21, IL-22, IL-23A, IFNA2, IFNG, TGFB2 and TNF, and the transcription factors (8), EOMES, FOXP3, GATA3, IKZF2, NRP1, RORA, RORG and TBX21) and ELISA (n = 72, TT=10, BT=10, BB=12, BV=8, VV=9, RT1=10 and RT2=13 - IFN-γ, IL-4 , IL-17, IL-6, TGF-β, IL-22 and IL-10. And for anti-PGL-1 (n = 75 – TT=9, BT=12, BB=12, BV=10, VV=8, RT1=11 and RT2=13).
PRAKOESWA et al., 2022	N=66 (33 mothers and 33 children)<br><br>Leprosy Spectrum ND.	BLOOD;<br>ELISA (IFN-γ, IL-4, IL-17 Th17 and FOXP3+).
SAINI et al., 2022	N=40<br><br>RT1=20 and NR=20	SKIN/BLOOD;<br>IMMUNOHISTOCHEMISTRY (skin - IL-21), Cells stimulated with MLSA, PHA and recombinant IL-21, ELISA (IL-17A/F, IL-21 and TGF-β), PCR (blood/skin - IL-21, TGF-β, IL-17A, IL-17F, RORC, FOXP3, and IFN-γ) and FLOW CYTOMETRY (blood - CD3, CD4, CD25, IL-17A, IL-21, FOXP3, and TGF-β).
KURIZKY et al., 2021	Case control female, 39 years old.	BLOOD;<br>FLOW CYTOMETRY (IFN-γ, TNF-α, IL-17A, IL-12/23p40, IL-10, IL-6, IL-4 and IL-2).

	BL/TIR (refractory neuritis)	
SAINI et al., 2018	N=40<br><br>BT=10, BT/RT1=10, LL=10, LL/RT2=10	BLOOD;<br>Cells stimulated with MLSA, FLOW CYTOMETRY (IFN-γ, IL-17, TGF-β, FOXP3, STAT3, STAT5, CD3, CD4, γδ-TCR and αβ-TCR), PCR (primers CD3γ, FOXP3, IL-17 , IFN-γ and TGF-β), ELISA (FN-γ, IL-17, IL-23, IL-1β and TGF-β).
HOOIJ et al., 2021	First Cohort n=155:<br>HC+BCG=50, HHC=54, EC=51<br><br>Second Cohort n=32:<br>HC+BCG=16, HHC=16<br><br>PB=7/MB=1 (developed leprosy during the 5-year follow-up).	BLOOD;<br>Sample with WCS, ELISA (GF, ENA-78 (CXCL5), Eotaxin (CCL11), FGF, Flt3L, Fraktalkine (CX3CL1), G-CSF, GM-CSF, GRO (CXCL1), GZMA, GZMB, I309 (CCL1) , sICAM1, IFN-α2, IFN-γ, IL-1α, IL-1β, IL-1rα, IL-2, IL-3, IL-4, IL-5, IL-6, IL-7, IL-8 , IL-9, IL-10, IL-12(p40), IL-12(p70), IL-13, IL-15, IL-16, IL-17A, IL17F, IL-20, IL-21, IL -22, IL-23, IL-27, IL-28A, IL-33, IP-10, MCP-1 (CCL2), MCP-3 (CCL7), MDC (CCL22), MIP-1α (CCL3), MIP -1β (CCL4), MMP2, MMP9, Myoglobin, PDGF-AA, PDGF-AB/BB, PRF, P-selectin, RANTES (CCL5), SAA, SAP, SCF, SDF-1, TGF-α, TNF-α , TNF-β, TPO, TRAIL, TSLP, sVCAM1 and VEGF.CCL17, I309, IL-3, IL-16, IL-17F, IL-20, IL-21, IL-22, IL-23, IL-27 , IL-28A, IL-33, SCF, SDF-1 and TSLP measured in the cross-sectional cohort. C1q, C3β, CFH, GZMA, GZMB, sICAM1, MMP2, MMP9, Myoglobin, PRF, P-selectin, SAA, SAP and sVCAM1 measured in the longitudinal cohort. PGL-I).
TARIQUE et al., 2017	N=50<br><br>BT=20, BL/LL=20, and HC=10	BLOOD;<br>Culture of PBMS stimulated with rIL-2, anti-CD3/CD28, plus recombinant IL-12 protein, IL-23, to reduce FoxP3 expression, or MLCwA with rIL-12 and rIL-23, FLOW CYTOMETRY surface: CD4, CD25, CD80, CD11c and CD86 / intracellular staining: FoxP3, pSTAT-3, IL-17A, IFN-γ, TGF-β and IL-10), ELISA (IFN-γ, TGF-β, IL- 10 and IL-17A) and WESTERN BLOTTING (Stat4).
SAINI et al., 2020	N=36<br><br>BT=18 and RT1=18	SKIN/BLOOD;<br>PCR (IL17A, IL17F, chemokines CCL1, CCL2, CXCL2, CXCL5, CCL7, CCL20, CXCL6, CXCL8, CXCL1, CXCL12, CX3CL1, and CCL22), Culture of cells stimulated with MLSA and PHA, FLOW CYTOMETRY (IL-17A and IL-17F) and ELISA (IL17A/F).
QUARESMA et al., 2015	N=50<br><br>I=10, TT=16, BB=17 and LL=7	SKIN;<br>IMMUNOHISTOCHEMISTRY (TGF-b, IL-6, IL-17).
DANG et al., 2019	N=16<br><br>BT=10 and LL=6	SKIN;<br>PCR (IL-26), cells stimulated with IL-26, IMMUNOFLUORESCENCE (IL-26, CD4, CD8, CD68).
FARAG et al., 2022	N=89<br><br>TT=15, BB=20, LL=25 and HC=29	BLOOD;<br>ELISA (IL-17A) and PCR (IL-17A single nucleotide polymorphism).
CASTRO et al., 2022	N=52<br><br>RT1/BL=10, BL=10, RT2/LL=9, LL=8, HC=15	BLOOD;<br>Culture of cells stimulated with MLSA and PHA, FLOW CYTOMETRY (CD4, CD8, CD2, FOXP3, IL-10 APC and TGF-β); ELISA (IFN-γ, IL-17, IL-23, IL-6 and IL-10).
BEZERRA-SANTOS et al., 2018	N=87<br><br>PB=39, MB=17 and HHC=31	BLOOD;<br>Cells incubated with MLCS, PPD, PHA and ML2028 recombinant protein; FLOW CYTOMETRY (TT=5, VV=6, HHC=6, HCT=6 - CD3, CD4, CD8, IL-2, TNF-α, and IFN-γ), Luminex<br>(IL-2, TNF-α and IFN-γ, IL-10 and IL-17A).
CHAITANYA et al., 2012	N=195<br><br>RT1=80, RT2=21,<br><br>NR=80 (TT=2, BT=58, BL=15, LL=2 and HPN=3)<br><br>HC=94	BLOOD;<br>ELISA (IL-17F)

VILANI-MORENO et al., 2021	N=45<br><br>RT2=14 (LL=7 and BL=7)<br>MB=16 (LL=7 and BL=9)<br>HC=15<br>Study limitation: 2 patients had recurrent RT2 and 1 died, they were excluded from the evaluation in M1, reducing from 14 to 11 patients in M1.	BLOOD;<br>FLOW CYTOMETRY (IL-2, IL-4, IL-6, IL-10, IL-17, IFN-γ, TNF), colorimetric Griess reaction (NO), enzyme-linked immunosorbent assay (PGL-1).
NEGERA et al., 2017	N=77<br><br>RT2=46 and LL=31	BLOOD;<br>FLOW CYTOMETRY (cell surface markers: CD3, CD4 or CD8, CD25, CD127, CD161 and FoxP3 were used to define regulatory T cells).
NEGERA et al., 2018	N=60<br><br>RT2=30 and LL=30	SKIN/BLOOD;<br>Cells incubated with MLCS, ELISA and PCR (TNF, IFN-γ, IL-β, TGF-β, IL-17A, IL-6, IL-8 and IL-10)

ND- Not described, HD- healthy donors, TT- tuberculoid, LL- Lepromatous, BT- borderline tuberculoid, RT1- type 1 reaction, RT2- type 2 reaction, MLSA- *M. leprae* sonicated antigen, NR- patients with non-reactive leprosy, PNL- pure neural leprosy, HCT- healthy control, BB– borderline borderline, BL- borderline lepromatous, PHA- phytohaemagglutinin, BCG- Bacillus Calmette-Guerin, WCL– *M. leprae* antigen; I= indeterminate, PBMC- peripheral blood mononuclear cells, EC- healthy individuals from the same area without contact with leprosy, WCS- *M. leprae* whole cell sonicate, HHC- healthy household contacts, MB- multibacillary, PB- paucibalillary, MLCS - *M. leprae* crude sonicated, PPD- Purified Protein Derived and NR- cases without reaction.
